# The Biomineralization of a Bioactive Glass-Incorporated Light-Curable Pulp Capping Material Using Human Dental Pulp Stem Cells

**DOI:** 10.1155/2017/2495282

**Published:** 2017-01-23

**Authors:** Soo-Kyung Jun, Jung-Hwan Lee, Hae-Hyoung Lee

**Affiliations:** ^1^Department of Biomaterials Science, College of Dentistry, Dankook University, Cheonan, Republic of Korea; ^2^Institute of Tissue Regeneration Engineering, Dankook University, Cheonan, Republic of Korea

## Abstract

The aim of this study was to investigate the biomineralization of a newly introduced bioactive glass-incorporated light-curable pulp capping material using human dental pulp stem cells (hDPSCs). The product (Bioactive® [BA]) was compared with a conventional calcium hydroxide-incorporated (Dycal [DC]) and a light-curable (Theracal® [TC]) counterpart. Eluates from set specimens were used for investigating the cytotoxicity and biomineralization ability, determined by alkaline phosphatase (ALP) activity and alizarin red staining (ARS). Cations and hydroxide ions in the extracts were measured. An hDPSC viability of less than 70% was observed with 50% diluted extract in all groups and with 25% diluted extract in the DC. Culturing with 12.5% diluted BA extract statistically lowered ALP activity and biomineralization compared to DC (*p* < 0.05), but TC did not (*p* > 0.05). Ca (~110 ppm) and hydroxide ions (pH 11) were only detected in DC and TC. Ionic supplement-added BA, which contained similar ion concentrations as TC, showed similar ARS mineralization compared to TC. In conclusion, the BA was similar to, yet more cytotoxic to hDPSCs than, its DC and TC. The BA was considered to stimulate biomineralization similar to DC and TC only when it released a similar amount of Ca and hydroxide ions.

## 1. Introduction

Bioactive glass-based materials have been introduced as promising hard/pulp tissue regenerative materials in dental fields, not only because of their apatite-forming ability, but also for their biomineralization ability for hard tissue (i.e., bone, enamel, dentin, and cementum) formation [1, 2]. To date, these materials have been applied to various types of dental materials, such as dental sealants/composite resins, bone or dental tissue scaffold matrices, and regenerative endodontic materials [3–8]. Recently, a bioactive glass-incorporated light-curable pulp capping material (pulp regenerative material, Activa™ Bioactive, Pulpdent, Watertown, MA, USA) has been introduced as the first bioactive dental product approved by the FDA in terms of bioactivity for indirect pulp capping materials; it provides quick setting times of less than 20 s for clinical convenience and biomineralization ability for hard tissue regeneration, and it has excellent biocompatibility [9].

Several in vitro and in vivo studies have evaluated the cytotoxicity, biocompatibility, or biomineralization ability of bioactive dental materials [10, 11]. Many studies have determined whether the mineral trioxide aggregate- (MTA-) like light-curable bioactive pulp capping materials can promote biomineralization to the same degree as conventional calcium hydroxide-incorporated chemically curable pulp capping materials, which have been considered the “gold standard” for approximately 20 years [12–14]. However, there is still a need for confirmatory testing with ionic supplement solutions that have the same amount of ions from extracts to confirm the role of biomineralization from released ions. In addition, there are newly developed bioactive glass-incorporated light-curable pulp capping materials that are still being investigated for their cytotoxicity and biomineralization compared with calcium hydroxide-incorporated chemically curable pulp capping materials [9].

Biomineralization, including alkaline phosphatase activity and mineral deposition from dental pulp stem cells (DPSCs), is an essential biofunctional characteristic of pulp capping materials [15]. When the dentin-pulp complex is damaged by dental caries or traumatic injuries, clinicians' efforts should allow the regenerated pulp tissue to be functionally competent, capable of forming mineralized tissue quickly to repair lost structure and to seal the clean pulp environment from the potentially contaminated external oral environment [16].

Reports have shown that isolated human DPSCs (hDPSCs) from extracted human teeth can be induced to differentiate into odontoblast-like cells in odontoblastic differentiating media and produce dentin-like mineral structures in vitro [17]. Therefore, they are widely used in investigations of dentin-pulp regeneration due to their clinical mimicking of pulp origin and their easy accessibility, which allows them to be readily isolated from extracted teeth [18–20]. However, to the best of our knowledge, no studies have been carried out to determine whether the newly developed bioactive glass-incorporated light-curable pulp capping material can promote biomineralization of hDPSCs or to determine the key mediators (possibly ions) required to induce biomineralization.

Therefore, the aim of this study was to perform a comparison of the cytotoxicity and biomineralization capability of the newly developed bioactive glass-incorporated light-curable pulp capping material with the calcium hydroxide-incorporated chemically curable product as the calcium releasing gold standard control and the MTA-like incorporated light-curable pulp capping material as the light-curable counterpart. In addition, the key players (ions) necessary for biomineralization induction were studied to investigate the underlying mechanism.

## 2. Materials and Methods

### 2.1. Pulp Capping Materials

Three indirect pulp capping materials were selected for this study: Activa Bioactive (BA, Pulpdent, Watertown, MA, USA) was chosen as a commercially available bioactive glass-incorporated light-curable pulp capping material, while Dycal® (DC, Dentsply, Milford, DE, USA) and Theracal LC® (TC, Bisco, Schaumburg, IL, USA) were selected as control calcium hydroxide-incorporated and light-curable pulp capping materials, respectively. The components and manufacturers of each pulp capping material are reported in Table 1. Each pulp capping material was applied in a Teflon mold, which was 10 mm in diameter and 2 mm in height. TC was dispensed from a syringe and applied in two layers of 1 mm in depth, and each layer was light cured with an LED light curing instrument (Litex 695, Dentamerica Inc., Industry, CA, USA) for 20 s according to the manufacturer's instructions (curing 1 mm depth per 20 s of light curing). BA was mixed using the dispensing syringe, added to the mold, and cured for 20 s using a curing light (Litex 695) according to the manufacturer's instructions (light curing setting time of 20 s for 4 mm depth of cure). Chemically self-cured calcium hydroxide liner (DC) was mixed according to the manufacturer's protocol, applied to the mold, and incubated at room temperature (23°C) with 50% relative humidity for 3.5 minutes (thermo-hygrometer, Daihan, Wonju, Korea). All procedures were performed on a sterilized clean bench to prevent any possible contamination. Each specimen from the mold was sterilized by ethylene oxide gas before further experiments.

### 2.2. Collection of Extract

Each pulp capping specimen was extracted at a ratio of 3 cm^2^/mL following the recommendations of ISO 10993-12 using distilled water (DW) [21]. Because the total surface area of the sample was 2.2 cm^2^, the samples were incubated in 0.73 mL of DW. To simulate the clinically adjustable environment, the eluates of all specimens were collected at 37°C for 24 hours in a shaking incubator (120 rpm).

### 2.3. Primary Culture of hDPSCs

The hDPSCs were from extracted third molars and were used after less than 10 passages throughout the experiments. Approval for their use was received from the institutional review board of Dankook University Dental Hospital (IRB number H-1407/009/004). Pulp tissues were antiseptically collected and added to phosphate-buffered solution (PBS) (Gibco, Grand Island, NY, USA) containing 1% penicillin/streptomycin (Gibco). After the addition of 0.08% collagenase type I (Worthington Biochemical, Lakewood, NJ, USA), the solution was incubated for 30 minutes with tapping every 10 minutes. Following enzymatic digestion, the hDPSCs were recovered by centrifugation at 1,500 rpm for 3 minutes. The cells were cultured in a humidified atmosphere of 5% CO_2_ at 37°C with supplemented media consisting of *α*-MEM with 10% fetal bovine serum (Gibco), 1% penicillin/streptomycin (Invitrogen, Carlsbad, CA, USA), 2 mM GlutaMAX (Gibco), and 0.1 mM L-ascorbic acid (Sigma Aldrich, St. Louis, MO, USA). All culture systems adhered to the above conditions. The stem cell functionality of hDPSCs was confirmed by positive expression (over 90%) of CD73, CD105, and CD106, and negative expression (less than 1%) of CD34 and CD45 by flow cytometry (data not shown). To promote biomineralization of the hDPSCs, we cultured the cells in odontogenic supplemental medium (OM) that included 50 *μ*g/mL ascorbic acid, 100 nM dexamethasone, and 10 mM *β*-glycerophosphate, as described previously [22].

### 2.4. Cell Viability

The cell viability test was performed according to ISO 10093-5 [23]. Briefly, 100 *μ*L of 1 × 10^5^ cells/mL were cultured in each well of a 96-well plate (SPL Life Sciences, Pocheon, Gyeonggi-do, Korea) with supplemented media (described in Section 2.3) in a humidified atmosphere of 5% CO_2_ at 37°C for 24 hours. After being washed with PBS (200 *μ*L), the cells were cocultured with 50 *μ*L of supplemented media and 50 *μ*L of extract or serially diluted extract by DW for another 24 hours. The percentages of the final concentrations of extract in the culture media were 50%, 25%, 12.5%, 6.25%, and 3.125%, and a mixture of 50 *μ*L of DW with 50 *μ*L supplemented media was used as the control (0%).

Cell viability was assessed with the MTS assay (CellTiter 96 Aqueous One Solution Cell Proliferation Assay, Promega, Madison, WI, USA) according to the manufacturer's protocol, and the results are expressed as the optical density percentage of each test group (*n* = 6) compared with each control group (*n* = 6). The optical absorbance was measured by a microplate reader (SpectraMax M2e, Molecular Devices, Sunnyvale, CA, USA) at a wavelength of 490 nm. To determine the toxicity of the tested pulp capping materials, a live-dead assay was performed. After the culture medium was removed, the cells were rinsed with PBS and incubated for 30 minutes with calcein AM (0.5 *μ*M) and ethidium homodimer-1 (4 *μ*M, Molecular Probes, Eugene, OR, USA). Images of live and dead cells were acquired using confocal laser scanning microscopy (CLSM, LSM700, Carl Zeiss, Thornwood, NY, USA) to confirm the above cell viability data. Live cells were identified by the presence of intracellular esterase activity, determined by the enzymatic conversion of the nonfluorescent cell-permeable calcein AM to green fluorescent calcein, which is retained within live cells. Dead cells were identified as stained with red fluorescent ethidium homodimer-1, indicating the loss of cellular membrane integrity. All analyses were independently performed in triplicate, and the representative means ± standard deviations (SD) or images are shown.

### 2.5. Alkaline Phosphatase Assay

Biomineralization was firstly estimated using an alkaline phosphatase (ALP) activity assay. ALP is an early biochemical marker for biomineralization. To measure ALP activity, we incubated noncytotoxic diluted extract (12.5%) with 1.2 mL of 10^5^/mL of hDPSCs in each well of a 12-well plate containing supplemented media or OM. Every 3 days, the media was replaced with fresh diluted extract (12.5%) conditioned OM, with the addition of DW (12.5%) for the positive control and the addition of DW (12.5%) to the supplemented media for the negative control. At days 14 and 21, the medium was removed, and 1 mL of 0.2% Triton X-100 (Sigma Aldrich, St. Louis, MO, USA) was added to each well. After the cell lysates were placed in a 1.5-mL centrifuge tube, the samples were processed through three freeze-thaw cycles (−70°C and room temperature, 45 minutes each) to rupture the cell membranes and extract the proteins and DNA from the cells, which were then centrifuged for 20 minutes at 13,000*g* at 4°C. Using the supernatant, alkaline phosphatase (ALP) activity (*n* = 6) was measured using p-nitrophenyl phosphate (at a final concentration of 20 mM, Sigma Aldrich) as the substrate in 1.5 M 2-aminomethyl-1-propanol (pH 10.3, Sigma Aldrich) and 1.0 mM MgCl_2_ (Sigma Aldrich). The absorbance at 405 nm was measured with a plate reader (SpectraMax M2e) and was normalized to the dsDNA quantity measured by a PicoGreen assay (*n* = 6); dsDNA from the supernatant was quantified using the Quant-iT PicoGreen Kit (Invitrogen) following standard protocols. Briefly, 100 *μ*L of each cell lysate solution was added to 100 *μ*L of PicoGreen reagent and incubated in the dark at room temperature for 5 minutes. The absorbance was read at an excitation/emission of 480/520 nm on a plate reader (SpectraMax M2e). All analyses were independently performed in triplicate, and the representative means ± SD are shown.

### 2.6. Alizarin Red Staining

Alizarin red staining (ARS) staining was performed to determine the mineralization ability of the noncytotoxic diluted extract (12.5%) using 1.2 mL of 10^5^/mL hDPSCs in each well of a 12-well plate. Every 3 days, the media was replaced with fresh diluted extract (12.5%) conditioned OM; then, DW (12.5%) was added to the OM as a positive control, and DW (12.5%) was added to the supplemented media as a negative control. After 21 days of incubation with supplemented media or OM, with the media being changed every 3 days, the cells were rinsed, fixed with 10% formaldehyde for 30 minutes, and stained with 40 mM alizarin red S (pH 4.2) for 30 minutes. After staining, the morphology was observed using light microscopy (Olympus lX71, Shinjuku, Tokyo, Japan). Quantitative analysis was carried out after the addition of 10% cetylpyridinium chloride (Sigma Aldrich) in 10 mM sodium phosphate (pH 7.0) for destaining. The concentration of ARS was determined by an absorbance measurement at 562 nm on a microplate reader (SpectraMax M2e). All analyses were independently performed in triplicate, and representative means (*n* = 5) ± SD or images are shown.

### 2.7. Detection of Calcium and Silicon Ions

The release of calcium (Ca), silicon (Si), or other possibly detected cationic ions (Zn, Ba, Na, Ti, and Fe) according to the composition of the products was measured from the extract with inductively coupled plasma atomic emission spectrometry (ICP-AES) (Optima 4300DV, Perkin-Elmer, Waltham, MA, USA), according to a previous protocol [24]. The detection limit was determined to be 0.1 ppm for all cations. To measure the released OH ions, the pH of the original extract and diluted extract in 12.5% with *α*-MEM were measured using a digital pH meter (Orion 4 Star, Thermo Scientific Pierce, IL, USA). Three measurements (*n* = 3) in each sample were performed, and all analyses were independently performed in triplicate to confirm reproducibility. The representative means (*n* = 3) ± SD were recorded.

### 2.8. Biomineralization from Released Ions

To prepare the ionic supplement added to BA, the ions that were differentially released between the TC and BA extract were added to the BA extract using CaCl_2_ (Sigma Aldrich) and NaOH (Sigma Aldrich) to match the ionic conditions of TC, which had 110 ppm Ca ions and pH 11. After the ionic supplement was added to BA, both BA and TC were diluted to 12.5% with OM to obtain a noncytotoxic extract, and they were cultured in OM for 21 d. DW diluted to 12.5% with OM was added to the culture with or without OM as negative and positive controls, respectively. The ionic supplement added to DW under the OM culture condition was used for investigating the effect of the ions released ions in the extract on the biomineralization of hDPSCs. Calcium deposition was measured using alizarin red staining (ARS) according to the above ARS section. After ARS was observed under the microscope, 10% w/v cetylpyridinium chloride monohydrate was used for quantitative analysis. The measurements were independently performed in triplicate, and representative averages (*n* = 5) with SD or images are presented.

### 2.9. Statistical Analysis

All experiments were independently carried out in triplicate to confirm reproducibility. The data were analyzed using one-way analysis of variance (ANOVA), followed by Tukey's post hoc test. The level of significance was *p* < 0.05.

## 3. Results

### 3.1. Cell Viability

**Figure 1:**
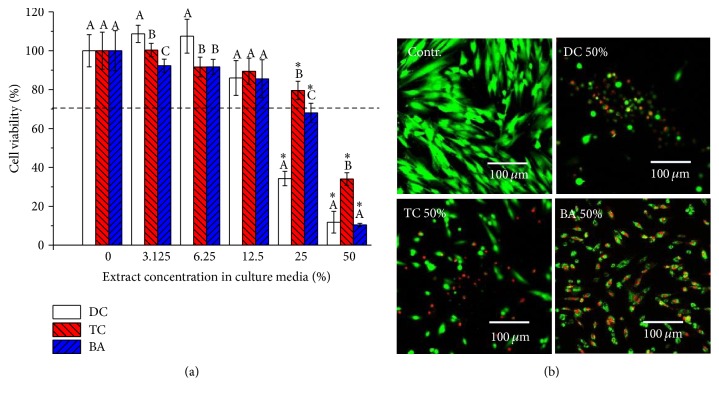
The cell viability results (a) and confocal microscopic images of live and dead cells (b) for Dycal (DC, control for the gold standard of pulp capping material), Theracal (TC), and Activa Bioactive (BA) stratified by serial dilution (0, 3.125, 6.25, 12.5, 25, or 50%) in culture with hDPSCs. Different letters indicate significant differences among the pulp capping materials in the same diluted extract culture (*p* < 0.05). The asterisk indicates a significant difference compared to the control (0%) for the extract from each product (*p* < 0.05). The dotted line shows 70% cell viability. Live cells (green) and dead cells (red) were observed using confocal microscopy. Images of live (green) and dead (red) cells in media supplemented with 50% distilled water (DW) as the control, DC, TC, or BA. The representative mean ± standard deviation (*n* = 6) and images are shown for experiments independently performed in triplicate.

The MTS assay results are reported in Figure 1. With incubation in the 50% extract, all groups showed less than 70% cell viability, which was significantly less than the control (0%). In particular, DC and BA had the lowest cytotoxic effect (almost 10% cell viability) among the groups (Figure 1(a), *p* < 0.05). With incubation in the 25% extract, all groups showed significantly less cell viability than the control (0%, Figure 1(a), *p* < 0.05), and DC had less than 70% cell viability. The 12.5%, 6.25%, and 3.125% groups were not significantly different compared to the control (Figure 1(a), *p* > 0.05). The CLSM images obtained after incubation in 50% extract in the supplemented media with different pulp capping materials confirmed these cell viability results. The results are shown in Figure 1(b). A significant number of dead cells (red) and few live cells (green) appeared in the DC, TC, and BA groups, while the control groups showed only live cells.

### 3.2. Biomineralization

**Figure 2:**
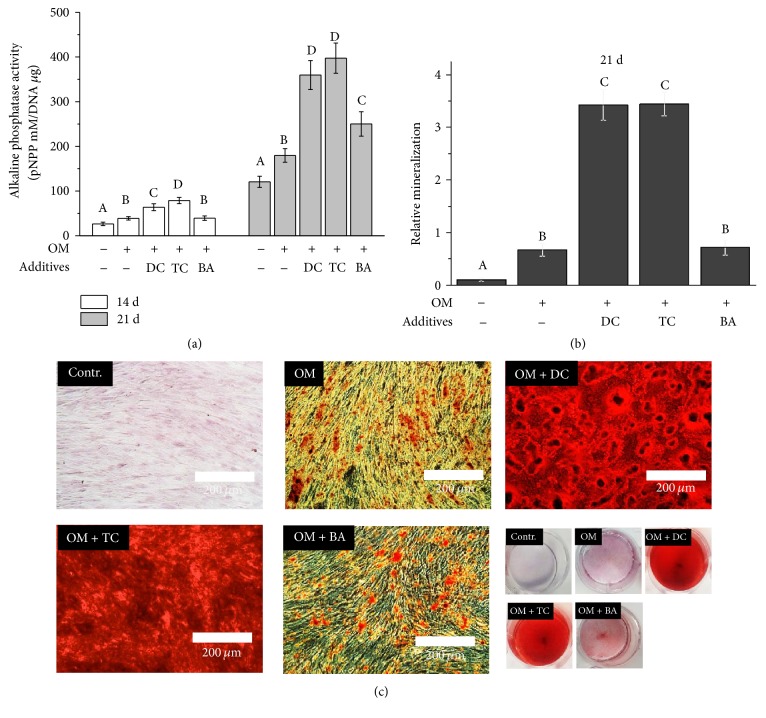
The effects of extracts from light-curable pulp capping materials (TC and BA) as well as the gold standard pulp capping material (DC) on alkaline phosphatase (ALP) activity (at 14 and 21 days) (a) and on calcium deposition using alizarin red staining (ARS) (at 21 days) (b and c). After ARS was observed under the microscope (c), 10% w/v cetylpyridinium chloride monohydrate was used for quantitative analysis (b). The cells were incubated for 7 to 21 days with 12.5% diluted extract in odontogenic supplemental medium (OM) for the differentiation of hDPSCs or supplemented media only for the negative control. Different letters indicate significant differences among the groups at *p* < 0.05. The measurements were performed in triplicate, and representative averages (*n* = 5) with SD or images are presented.

ALP activity from the “pulp capping material extract”-treated cells was minimal on day 14 and increased on day 21 (Figure 2). At 14 and 21 days of culture, statistically significant increases in ALP activity were noted in the DC and TC extract (12.5%) treated cells compared with OM-treated control cells (Figure 2(a), *p* < 0.05), but this difference was only observed at 21 days of culture in the BA extract (12.5%). At 21 days of culture, the DC and TC groups had significant ALP activity compared to the BA group (Figure 2(a), *p* < 0.05). Moreover, there was a clear difference in the ARS-stained images from the DC and TC groups compared to the BA- or OM-treated control cells, and quantification of the eluted products revealed a statistically significant difference in the DC and TC groups compared to the other groups (Figures 2(b) and 2(c), *p* < 0.05).

### 3.3. Biomineralization of the Released Ions

**Figure 3:**
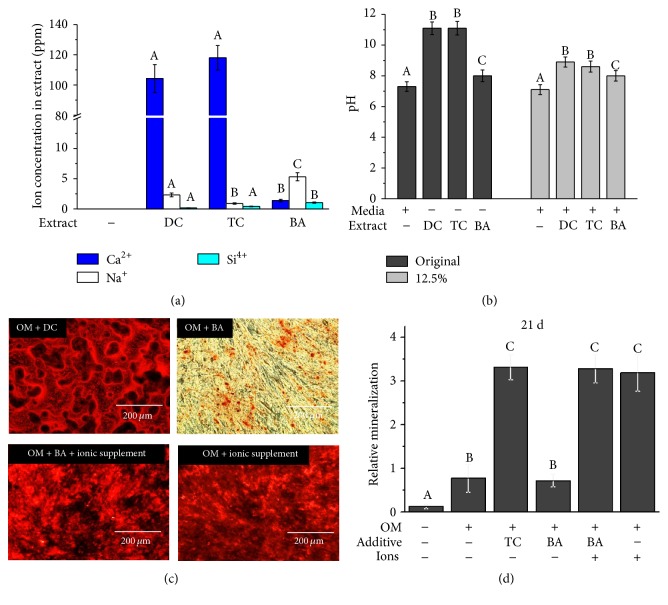
The ions released from the extracts of pulp capping materials as detected by inductively coupled plasma atomic emission spectrometry analysis (a) and pH measurement (b). (c and d) After an ionic supplement including the ions that were differentially released between the TC and BA extract was added to the BA extract to match the ionic conditions, the ionic supplement-added 12.5% BA extract was cultured with odontogenic supplemental medium (OM) for 21 days to compare the biological effects of 12.5% TC, BA, and ionic supplement. Calcium deposition was measured using alizarin red staining (ARS). After ARS was observed under the microscope (c), 10% w/v cetylpyridinium chloride monohydrate was used for quantitative analysis (d). The measurements were performed in triplicate, and representative averages (*n* = 5) with SD or images are presented. Different letters indicate significant differences among the groups at *p* < 0.05.

The release of Ca and OH ions was observed to be significantly higher in the DC and TC groups compared to the BA group (Figures 3(a) and 3(b), *p* < 0.05). Other ions, such as Zn, Ba, and Ti, were detected at less than 1 ppm or were not detected. Si and Na ions were detected in all groups but at levels less than 1.5 and 6 ppm, respectively. Ionic supplement-conditioned BA had statistically similar mineralization ability compared to the DC and ionic supplement only groups under OM culture (Figures 3(c) and 3(d), *p* > 0.05).

## 4. Discussion

Pulp capping materials have been shown to play a vital role as restorative materials in the successful regeneration of the dentin-pulp complex [3, 25, 26]. Pulp capping materials have not only pulp sealing effects but also biological properties such as biomineralization, which leads to dentin-pulp complex regeneration [25]. Recently, a bioactive glass-incorporated light-curable pulp capping material was released to the market, but investigations of the biological activities in mammalian cells that result from its use are limited. After evaluating the cytotoxicity compared with calcium hydroxide-incorporated and MTA-like light-curable pulp capping materials, we investigated the biomineralization of a commercially available bioactive glass-incorporated light-curable pulp capping material to determine its potential as a dentin-pulp regenerative material.

### 4.1. Cytotoxicity

First, the elutes were serially diluted with DW and added to the culture media for 24 hr. The method by which pulp capping materials meet the hDPSCs is via the extract through dentinal tubules, not by direct contact. Therefore (diluted) extract was added to hDPSCs, and cytotoxicity was evaluated. Cytotoxicity over 30% (cell viability less than 70%) was revealed in the 50% culture conditions in all experimental groups and in the 25% culture conditions for DC, most likely due to the alkaline pH (>10) and high concentrations of ions. The calcium hydroxide-incorporated (DC) and MTA-like light-curable (TC) pulp capping materials showed excellent biocompatibility according to in vivo and clinical studies, but they also showed in vitro cytotoxicity in these results. Therefore, we concluded that the newly developed bioactive glass-incorporated light-curable pulp capping material (BA), which revealed similar or greater in vitro cytotoxicity compared to both DC and TC, has relatively biocompatible characteristics. Most of light curing materials including BA are polymerized from monomers with the help of a photoinitiator such as camphorquinone, and when these components are released into the applied tissue, they can be toxic to the surrounding cells. According to the components in BA listed in the MSDS and the polymerizing mechanism, unpolymerized monomers, silica, fluoride ions, and camphorquinone could be among the reasons for the induction of cytotoxicity. Further studies investigating the releasable cytotoxic inducers are needed to comprehend the cytotoxicity of BA. To exclude the potential adverse effect on biomineralization from the cytotoxicity-induced biological cascades, which negatively affect the therapeutic characteristics, a 12.5% culture condition was chosen, which showed less than 30% cytotoxicity in all tested groups at the first time point.

### 4.2. Biomineralization of hDPSCs

ALP activity and ARS staining, which are well-known biomolecule assays for investigating biomineralization [27], were detected at significant levels in the TC and DC groups compared with the OM-treated control group (*p* < 0.05) but were not significantly expressed in the BA group (*p* > 0.05). ALP activity and ARS are correlated with calcium-phosphate matrix formation in dentin prior to the initiation of mineralization [28]. High ALP activity provides high concentrations of phosphate at the site of mineral deposition. ARS is used to quantitatively determine calcific deposition by cells, which is a crucial step toward the formation of the calcified extracellular matrix in a later stage. Differentiated odontoblasts from hDPSCs produce dentin, which consists of calcium and phosphate. Therefore, the above two assays were promising for the evaluation of biomineralization [3]. We concluded that the bioactive glass-incorporated light-curable bioactive pulp capping material has less potential for similar therapeutic effects in terms of biomineralization from hDPSCs compared to calcium hydroxide-incorporated and MTA-like incorporated light-curable pulp capping materials.

Direct comparisons among the above-evaluated products in terms of cytotoxicity and consequent biomineralization is beyond the scope of this discussion; however, compared with the calcium hydroxide-incorporated pulp capping material (DC), the MTA-like light-curable pulp capping material (TC) has shown comparable biocompatibility and biomineralization owing to released Ca and OH ions [29]. However, the bioactive glass-incorporated pulp capping material (BA) did not show comparable biomineralization, possibly due to a lack of released Ca and OH ions. In general, compared with the conventional calcium hydroxide-incorporated pulp capping material, light-curable bioactive pulp capping materials have quick setting times in light-activated systems, which are more clinically beneficial for the restoration of dentin and enamel on pulp capping materials. Of course, light-curable Dycal (Prisma VLC Dycal) could be used as control, but its biomineralization ability was considered less than that of the conventional chemical curing Dycal (DC) because released calcium ions were not detected [30, 31]. For use in base materials, bioactive light-curable pulp capping materials have advantages such as greater stability and mechanical properties [12, 31]. The major component of light-curable bioactive pulp capping materials is composite resin, which, when adjusted in dentin, has higher mechanical and chemical stability against internal and external stimuli, such as dentin fluid and biting force [32, 33]. Future in vitro and in vivo studies are needed to directly compare the in vivo biomineralization and sealing effects of calcium hydroxide-incorporated pulp capping materials and the two other tested types of pulp capping materials in dentinal tubules to use bioactive glass-incorporated light-curable pulp capping materials as a base and liner material in clinical settings.

### 4.3. Biomineralization from Released Ions

When extracts from pulp capping materials affect hDPSCs, the ions released in the extract are key players in the induction of biomineralization. It has already been revealed that extracellular Ca^2+^, Si^4+^, and OH^−^ combine to regulate the biomineralization of hDPSCs [21, 34]. In previous experiments, osteogenic or odontogenic biomineralization from stem cells was encouraged by the dissolution of ions (Si^4+^, Ca^2+^, and OH^−^) [35, 36]. Concentration of released Ca^2+^ and OH^−^ was significantly increased in the TC and DC treated groups compared to that from BA treated group. Even though Na^+^ and Si^4+^ were detected at significant levels of approximately 5 ppm and 1 ppm, respectively, in the BA group, these ions were not present at effective dosages for stem cells, including hDPSCs [37]. Therefore, to confirm the role of released ions in terms of biomineralization, an ionic supplement matching the ionic environment of TC with Ca^2+^ (110 ppm) and OH^−^ (pH 11) was added to the BA-treated group, and 12.5% diluted TC and BA, and ionic-supplemented BA were compared in terms of mineralization. The ionic supplement-conditioned BA and DW group under OM culture media had similar mineralization abilities compared to the TC group (*p* > 0.05), which suggested that the released ions played key roles in inducing biological properties in hDPSCs. By combining the above results with the data on the released ions, within the limitations of this study, it can be postulated that light-curable pulp capping materials stimulate biomineralization at the same level as that from the calcium hydroxide-incorporated gold standard materials when they release the same amount of Ca and OH ions. Of course, other in vitro assays, such as ALP activity and mRNA/DNA expression assays, are needed to confirm the essential role of Ca and OH ions for biomineralization. In addition, further in vivo studies of the biocompatibility and biomineralization, as well as mechanical/physical properties, such as compressive strength, microleakage, and biodegradability, are needed to clarify the suitability or performance ability of bioactive glass-incorporated light-curable pulp capping materials for clinical applications compared to calcium hydroxide-incorporated and MTA-like light-curable pulp capping materials.

## 5. Conclusions

Extracts from a bioactive glass-incorporated light-curable pulp capping material were evaluated for cytotoxicity and biomineralization of hDPSCs. The eluates from the bioactive glass-incorporated light-curable pulp capping material (BA) were similar to and more cytotoxic to hDPSCs than those from calcium hydroxide-incorporated (DC) and MTA-like incorporated (TC) pulp capping materials. In addition, the BA extract promoted biomineralization along with ALP activity and ARS staining compared to the OM-treated control. However, BA only showed similar mineralization after matching the ionic conditions to those of the other tested pulp capping materials. Therefore, within the limitations of this in vitro study, BA exhibited the potential to stimulate biomineralization at the same level as other pulp capping materials it released the same amount of Ca and OH ions. Further in vitro studies (odontogenic differentiation markers at the gene or/and protein level) and in vivo or clinical studies are necessary to compare the regenerative potential of BA compared to other pulp capping materials on pulp tissue.

## Figures and Tables

**Table 1:** The pulp-capping materials tested in this study.

Product	Code	Manufacturer	Lot number	System	Composition (wt%)^*∗*^
Dycal	DC	Dentsply, USA	150804	Self-curing (calcium hydroxide based)	Base paste: 1,3-butylene glycol disalicylate (<50%), calcium tungstate (<20%), zinc oxide (<15%), and so on
					Catalyst paste: calcium hydroxide (<55%), zinc oxide (<15%), titanium oxide (<10%), and so on
Theracal LC	TC	Bisco, USA	1500006479	Light curing (Portland cement based)	Portland cement type III (<60%), polyethylene glycol dimethacrylate (<50%), barium zirconate (<10%), and so on
Activa Bioactive™	BA	Pulpdent, USA	150814	Light curing (resin based)	Blend of diurethane and other methacrylates with modified polyacrylic acid (~53.2%), silica (~3.0%), sodium fluoride (~0.9%), and so on

^*∗*^Composition was filled with the manufacturers' available information including material safety data sheets.
